# Host specificity drives genetic structure in a freshwater mussel

**DOI:** 10.1038/s41598-019-46802-8

**Published:** 2019-07-18

**Authors:** Sebastian Wacker, Bjørn Mejdell Larsen, Sten Karlsson, Kjetil Hindar

**Affiliations:** Norwegian Institute for Nature Research (NINA), P.O. Box 5685 Torgarden, N-7485 Trondheim, Norway

**Keywords:** Evolutionary ecology, Coevolution, Genetic variation

## Abstract

Parasites often depend on their hosts for long distance transport, and genetic population structure can be strongly affected by host specificity and dispersal. Freshwater pearl mussel (*Margaritifera margaritifera*) populations have previously been found to naturally infest either primarily Atlantic salmon (‘salmon-mussel’) or exclusively brown trout (‘trout-mussel’) across a wide geographic range. Here, we experimentally test whether this intraspecific variation in natural infestation can be explained by host specificity in freshwater pearl mussel. Our experiments show that when both host species were exposed to larvae from salmon- and trout-mussel respectively, salmon-mussel larvae almost never infested brown trout and vice versa. This suggests that host specificity can explain variation in natural infestation among the studied freshwater pearl mussel populations. Host specificity provides a link to the species’ variable population genetic structure, as mussel populations limited to Atlantic salmon, the host with stronger dispersal, show higher genetic diversity and weaker differentiation than populations limited to brown trout as host.

## Introduction

Host specificity is important for major evolutionary processes in parasites, including speciation and extinction. Host switches in specialist parasites may promote speciation by adaptive radiation^1,2^ and specialist parasites are more prone to coextinction by the loss of suitable hosts^3,4^. Host specificity may also be an important determinant of the dispersal of parasites and their potential for colonisation. Many parasites spend parts of their lifecycle inside or attached to a host and depend on their host for the dispersal of gametes, progeny or adults^5^. For those species, dispersal and colonisation vary in dependence of the behaviour and mobility of the host, with concomitant consequences for genetic population structure and geographic distribution^6–8^. Knowledge of host specificity is therefore vital for the management of threatened parasite species, predicting their vulnerability to changes in host distribution and the adaptive potential and colonisation abilities of populations.

Freshwater mussels (Bivalvia: Unionoida) are among the most endangered animal taxa^9,10^. Their complex lifecycle includes a parasitic stage, during which larvae of most species live encapsulated on the gills or fins of a fish host, where they develop into juveniles. Host specificity varies widely among freshwater mussels, from specialists that are limited to a single fish species to generalists using more than 50 species^11^. Reproduction of the sedentary adults relies on the passive transport of sperm from the male to the female and of larvae from the female to the fish host. In rivers, such passive transport in the water column is limited to short distances downstream. Any upstream or longer-distance dispersal of freshwater mussels, including between-population gene flow and colonisation, is therefore dependent on the fish host^12–16^. As a consequence, species of freshwater mussels differ in genetic structure in dependence of the dispersal of their fish hosts^14,17,18^. However, little is known about variation in host specificity and its genetic consequences within species of freshwater mussels^11^.

Intraspecific variation in natural infestation of host fishes has been described across freshwater pearl mussel (*Margaritifera margaritifera*) populations from 25 localities in Norway^19^. Larvae were found almost exclusively on either Atlantic salmon (*Salmo salar*) or brown trout (*Salmo trutta*). When both host fishes were present, Atlantic salmon was strongly infested, while brown trout was only sporadically infested. At localities with brown trout only, brown trout was strongly infested. Similar patterns of host infestation have been found in other Northern European countries where both host species co-occur with freshwater pearl mussel^20–22^. Differences in host usage were tightly associated with the genetic structure of mussel populations in Norway^19^ and Ireland^21^. Populations parasitizing on Atlantic salmon (‘salmon-mussel’^19^) had a higher genetic diversity and weaker among-population differentiation than populations parasitizing on brown trout (‘trout-mussel’^19^). Those patterns were found across populations in different rivers, but also within the same river, and may be explained by differences in host fish dispersal.

Atlantic salmon may form genetically distinct populations within and between rivers, but some dispersal occurs between rivers^23,24^. The trout-mussel populations described by Karlsson *et al*.^19^ are with one exception located upstream from natural barriers for fish migration (waterfalls and rapids), preventing ongoing dispersal between populations of non-anadromous brown trout (‘landlocked brown trout’ hereafter) and the associated mussel populations between rivers. Dispersal of Atlantic salmon and isolation of landlocked brown trout are expected to result in stronger gene flow among salmon- than trout-mussel populations, and a weaker genetic differentiation. However, it remains unknown to what extent the observed biased infections on fish gills across Norwegian populations are caused by host specificity alone or are confounded by other factors in the natural environment. Infestation in the wild may for example be affected by fish behaviour if brown trout and Atlantic salmon seek different microhabitats and thereby differ in exposure to mussel larvae. Field observations of live infested fish may not be representative for infestation success if infestation affects fish mortality and differently so in the two host species. Furthermore, it is largely unknown if host specificity in trout-mussel applies differently to landlocked brown trout and anadromous brown trout (sea-run migratory), because most of the studied trout-mussel populations co-occur with landlocked brown trout only and their potential for infesting Atlantic salmon and anadromous brown trout is unknown.

**Figure 1:**
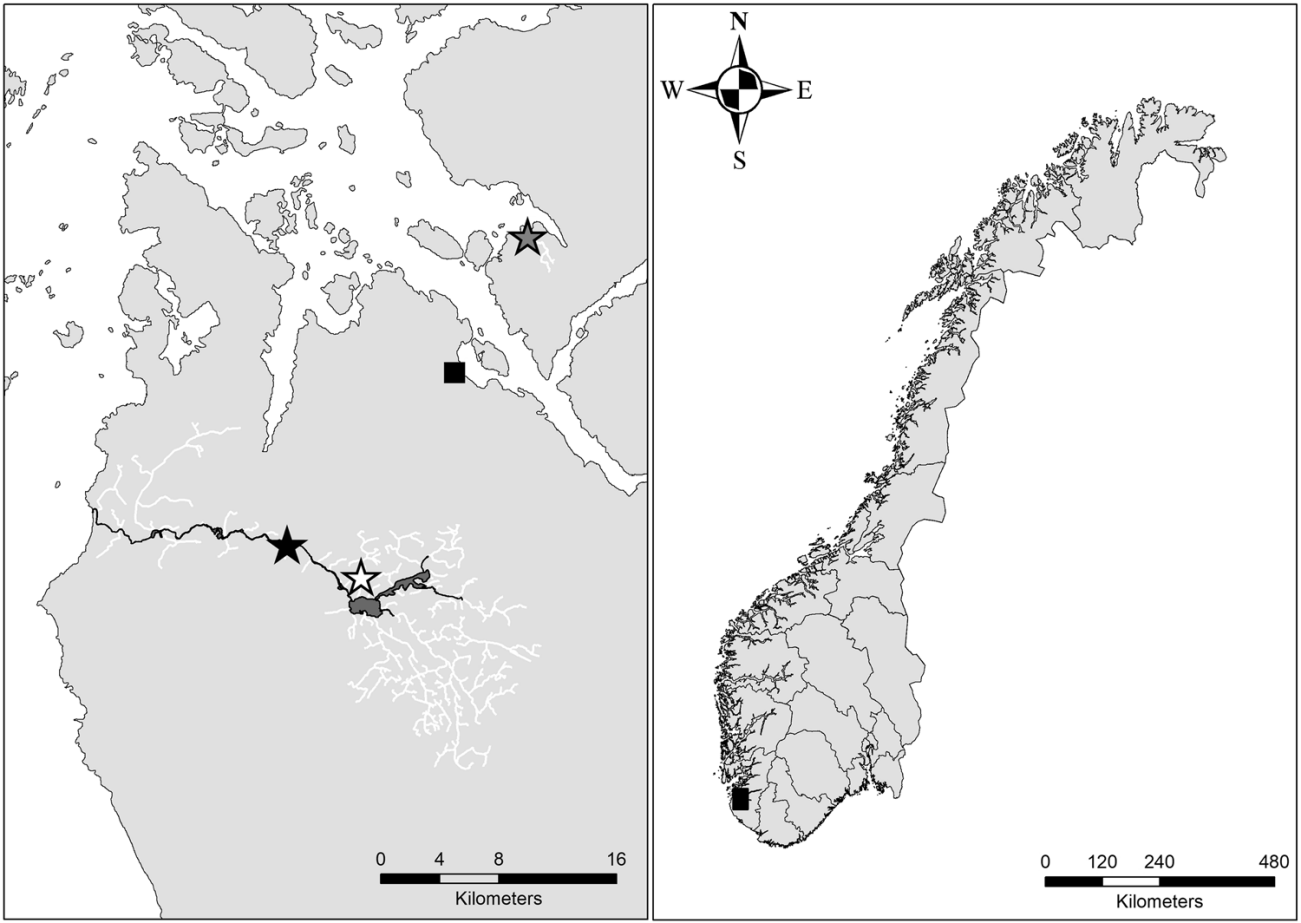
Location of the studied freshwater pearl mussel populations and the NINA Research Station Ims (black square). *River Flotåna* (white star) and *River Svinesbekken* (grey star) are trout-mussel populations, while *River Figgjo* (black star) is a salmon-mussel population. The anadromous section of the *River Flotåna* system is highlighted dark. The area shown in detail is marked black in the map showing Norway.

In this study, we experimentally test for differences in host specificity between closely situated populations of salmon- and trout-mussel (Fig. 1). The studied salmon-mussel population co-occurs with both Atlantic salmon and anadromous brown trout and shows high genetic diversity and natural infestation on Atlantic salmon^19^. The two studied trout-mussel populations co-occur with landlocked brown trout only and show low genetic diversity and natural infestation on brown trout^19^. In the experiment, Atlantic salmon, anadromous brown trout, and landlocked brown trout were exposed to larvae from naturally fertilised salmon- and trout-mussel and infestation intensity was monitored over a period of four months. Infestation in small tanks ensured that all host fish were exposed to mussel larvae. Infestation of host fish in a common infestation bath and monitoring of infestation over an extended period follow recommendations for freshwater mussel infestation assays^25^. Our experiment allowed to test host specificity for salmon- and trout-mussel on the full range of potential hosts, including infestation of Atlantic salmon and anadromous brown trout by mussels that naturally co-occur with landlocked brown trout only. We tested the hypothesis that trout-mussel predominantly infest brown trout and salmon-mussel predominantly infest Atlantic salmon when both hosts are equally exposed to larvae.

## Results

**Figure 2:**
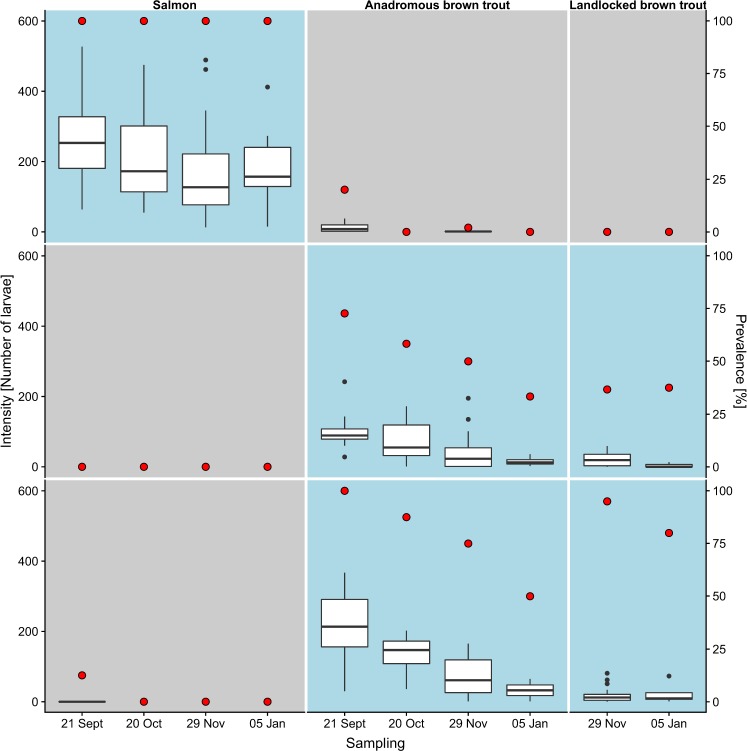
Infestation of host fishes (Atlantic salmon, anadromous brown trout, landlocked brown trout) by larvae from three Norwegian freshwater pearl mussel populations: *River Figgjo* (upper row), *River Flotåna* (mid row), *River Svinesbekken* (lower row). Infestation prevalence is given as the percentage of fish that was infested (red dots) and infestation intensity as the number of larvae on the left gills of infested fish (boxplots). Infestation was measured at four sampling occasions over a period of 15 weeks. Blue background colour indicates expected host specificity, based on infestation in the wild. Sample size varies between 5 and 50 for combinations of host fishes, mussel populations and sampling occasions. Box plots show medians, upper/lower quartiles, minima and maxima. Black dots show outliers.

Host infestation differed distinctly between the mussel population co-occurring with both Atlantic salmon and anadromous brown trout (*River Figgjo*) and the two mussel populations co-occurring with landlocked brown trout only (*River Flotåna* and *River Svinesbekken*) (Fig. 2). We found that salmon-mussel larvae almost exclusively infested Atlantic salmon, while trout-mussel larvae almost exclusively infested brown trout, regardless of whether they were anadromous or landlocked (Fig. 2). These differences were evident throughout the period of 15 weeks over which infestation was monitored (Fig. 2).

Infestations of Atlantic salmon by trout-mussel and vice-versa were extremely infrequent and only low numbers of larvae per fish were found in those instances (Fig. 2). Only one out of 176 Atlantic salmon was infested by trout-mussel larvae, and only five out of 110 brown trout were infested by salmon-mussel larvae. In addition, larvae from salmon-mussel infesting brown trout and vice-versa showed reduced growth compared to larvae infesting the expected hosts (Supplementary Fig. S1).

The trout-mussel populations in this study naturally co-occur with landlocked brown trout only, but successfully infested both landlocked brown trout and anadromous brown trout in our experiment (Fig. 2). Infestation was even slightly stronger on anadromous brown trout than on landlocked brown trout (Wilcoxon signed-rank test on within tank differences: V = 53.5, P = 0.009, N = 10 pairs; Supplementary Fig. S2). Anadromous brown trout carried higher numbers of larvae, despite being smaller than landlocked brown trout (TL sampled anadromous brown trout: 99.6 ± 7.8 mm, TL sampled landlocked brown trout: 152.0 ± 10.8 mm, mean ± SD; paired t-test: t = −10.0, P < 0.001, N = 10 pairs).

Infestation of Atlantic salmon remained high throughout the experiment. Infestation of anadromous brown trout decreased for both trout-mussel populations, but was within the range observed in natural populations^19^ by the end of the experiment (Fig. 2). On the first sampling occasion, a large proportion of anadromous brown trout was strongly infested by trout-mussels from *River Flotåna* (Fig. 2). On the third sampling occasion, there was large variation in the infestation of (anadromous and landlocked) brown trout among trout-mussels from *River Flotåna*, with two out of six mussels showing considerable infestation and four mussels showing only sporadic infestation (Supplementary Fig. S3).

## Discussion

We have demonstrated that populations of freshwater pearl mussel from a small geographic area differ fundamentally in host specificity. Mussels of the tested salmon-mussel population were limited to Atlantic salmon and mussels of the two trout-mussel populations were limited to brown trout as host, even when both species were abundant and in contact with parasitic mussel larvae. We further demonstrated that the species specificity in trout-mussel naturally co-occurring with landlocked brown trout also applied when a non-local landlocked brown trout population or anadromous brown trout was the host. These findings suggest that host specificity is primarily expressed at the species level. Our results add experimental evidence that variation in natural host infestation^19^ is explained by host specificity. Potentially confounding factors, such as differences in fish behaviour that affect their exposure to mussel larvae or differences in mortality between infested Atlantic salmon and brown trout, cannot explain our experimental results.

Intraspecific variation in host specificity has previously been found among freshwater pearl mussel populations in Finland^20^, but host specificity was less strongly expressed in Finnish trout-mussel populations than in our study. It remains to be tested whether Finnish trout- and salmon-mussel populations differ in genetic structure and whether Finnish trout-mussel populations successfully infest anadromous brown trout. Intraspecific variation in natural host infestation has recently also been reported for freshwater pearl mussel populations in Ireland, with western populations almost exclusively infesting Atlantic salmon and inland populations exclusively infesting brown trout^21^. In line with results from Norwegian freshwater pearl mussel populations^19^, Irish populations infesting Atlantic salmon had a higher genetic diversity and weaker among-population differentiation than populations parasitizing on brown trout^21^. Our results strongly suggest that the studied salmon- and trout-mussel populations differ in genetic structure because of different dispersal of their hosts. Salmon-mussel populations experiencing significant gene flow via straying Atlantic salmon show low genetic differentiation, while trout-mussel populations co-occurring with isolated, landlocked brown trout populations show strong genetic differentiation. Together with recent evidence of intraspecific variation in natural host infestation in Finland^20^ and Ireland^21^ and of concomitant variation in genetic population structure in Ireland, our results suggest that host specificity is a strong determinant of the genetic structure of freshwater pearl mussel across its Northern European distribution.

The genetic structure of salmon- and trout-mussel may not only be affected by host dispersal via between-population gene flow, but also via their postglacial colonisation history. Salmon- and trout-mussel may represent distinct evolutionary lineages, with independent postglacial colonisation histories, linked to the colonisation histories of their respective hosts^26^. Alternatively, salmon- and trout-mussel may have a common colonisation history, and their differentiation resulted from local adaptation after postglacial colonisation^27^. Although the latter scenario may intuitively appear more correct, the first scenario is more likely because genetic variation was not explained by geographical region and salmon- and trout-mussel populations within rivers were highly differentiated^19^. Strong infestation of anadromous brown trout and almost no infestation on anadromous Atlantic salmon by trout-mussel larvae from populations being isolated and landlocked since the last glaciation indicates a persistence to brown trout as the functional host, which is in line with the scenario of independent postglacial colonisation.

Our results add to previous knowledge on host specificity in freshwater mussels and its genetic consequences^11,28^. Infestation experiments have previously shown host specificity in freshwater pearl mussel for host species^29^, strains of the same host species^29,30^ and host fish age and condition^31–33^. Species of freshwater mussels express host specificity for different host species and differences in the dispersal of the fish hosts affect the genetic population structure of the mussel parasite^14,17,18^. Our results show that variation in host specificity also occurs among populations of the same freshwater mussel species and within a small geographic scale. In line with our results, populations of the freshwater mussel *Unio crassus* have been found to differ in infestation success among host species and among host strains^34,35^. In contrast to our results and previous studies in Finland^20^ and Ireland^21^, two Swedish freshwater pearl mussel populations co-occurring with Atlantic salmon and brown trout only infested brown trout^36^. Future studies are needed to reveal whether intraspecific variation in host specificity is common in freshwater mussels and how this affects their genetic population structure.

Our study was limited to one salmon-mussel population and two trout-mussel populations, which almost exclusively infested their respective host species. Natural infestations of brown trout can be more common in other salmon-mussel populations, but infestation intensity is always very low compared to infestation of coexisting Atlantic salmon^19^. Salmon-mussel from other populations have been observed to infest brown trout in laboratory conditions in a Norwegian hatchery for freshwater pearl mussel (Per Jakobsen, University of Bergen, Norway, pers. comm.). However, further studies are needed to test whether those differences in host specificity are caused by higher infestation pressure or the use of domesticated strains of brown trout in the hatchery. Host specificity was also less pronounced in Finnish freshwater pearl mussel populations^20^ and a broader approach is needed to study geographic variation in the strength of host specificity, and its causes and genetic consequences in Norway and across the species’ distribution. Further studies are also needed to resolve the host specificity and genetic structure of freshwater pearl mussel co-occurring with anadromous brown trout, in the absence of Atlantic salmon. Only few such populations are known in Norway and the single population that was included in the genetic survey by Karlsson *et al*.^19^ showed genetic variation in-between typical trout- and salmon-mussel populations.

Our results suggest that intraspecific variation in host specificity can affect a parasite’s population genetic structure within a small geographic area, when hosts differ in dispersal abilities. This has important consequences for freshwater pearl mussel conservation. Freshwater pearl mussel has recently experienced dramatic declines across its historical distribution in Europe and is subject to extensive conservation efforts^10^. Trout-mussel populations may be more vulnerable due to lower genetic diversity and a highly limited potential for dispersal and colonisation^19^. Conservation units may also be defined on smaller geographical scale for trout-mussel, because of their higher genetic differentiation compared to salmon-mussel. The loss of suitable hosts is a major threat for European freshwater mussels^10^ and strong intraspecific variation in host specificity emphasises the importance of protecting the primary host fish for a given mussel population^10,19^. Across species, freshwater mussels have previously been found to have a better conservation status when their hosts showed strong dispersal^37^. Our findings emphasize the importance of intraspecific variation in host specificity and host dispersal for the conservation of globally endangered freshwater mussels.

## Methods

### Ethical statement

All experiments were carried out in accordance with relevant guidelines and regulations. The study was carried out with approval granted from the *Norwegian Food Safety Authority* for NINA Research Station Ims (approval *051*) and from the *Directorate of Fisheries* for the use of experimental fish (approval *R SS0020*).

#### Mussel populations

We tested host specificity in three populations of freshwater pearl mussel in SW Norway (*River Figgjo*, *River Flotåna* and *River Svinesbekken*; Fig. 1), differing in the natural occurrence of host species. The aim of this study was to compare host specificity of mussels co-occurring with both Atlantic salmon and brown trout with mussels co-occurring with landlocked brown trout only. This reflects the categories of salmon-mussel and trout-mussel populations that have been established on the basis of the natural infestation of host fishes^19^. *River Figgjo* (58°47′N, 5°46′E) is a large river with Atlantic salmon, anadromous brown trout and landlocked brown trout. *River Flotåna* (58°46′N, 5°51′E) and *River Svinesbekken* (58°59′N, 6°02′E) are small brooks with landlocked brown trout only. *River Flotåna* is a tributary to *River Figgjo*, and mussel populations are located above a migration barrier. *River Svinesbekken* is not connected to *River Figgjo* (ca 55 km shortest seaway between outlets; Fig. 1) and anadromous fish can probably only enter the lowermost part of *River Svinesbekken*, where no freshwater pearl mussel occur. Natural infestations are found on Atlantic salmon only in *River Figgjo*, and on landlocked brown trout only in *River Flotåna* and *River Svinesbekken* (Table S1 in Karlsson *et al*.^19^). Genetic diversity in the three populations followed the same pattern as has been shown for salmon- and trout-mussel populations, respectively, with higher heterozygosity and allelic richness in *River Figgjo* than in *River Flotåna* and *River Svinesbekken* (Table 1 in Karlsson *et al*.^19^).

#### Infestation assay

Naturally fertilised gravid mussels (i.e. females carrying larvae on their gills) were collected in the three populations between 7 and 12 August 2006 and transported separately to NINA Research Station Ims (Norway). At the research station, mussels were immediately placed individually into 2 m^3^ fibreglass tanks. Tanks were supplied with a continuous flow of water from River Imsa, following natural variation in temperature (range 3.1–18.9 °C^38^). We tested host preference for ten salmon-mussels (*River Figgjo*) and a total of ten trout-mussels (six *River Flotåna*, four *River Svinesbekken*). On 1 August 2006, 42–43 Atlantic salmon, 46–49 anadromous brown trout and 6–8 landlocked brown trout were introduced to each tank. All fish were first generation descendants from wild *River Figgjo* fish, bred at the research station. *River Figgjo* mussels were thus sympatric with the used host fishes, while *River Flotåna* and *River Svinesbekken* mussels were allopatric with the used brown trout. Numbers per host type and tank varied slightly due to availability. All fish were young-of-the-year (0+), the age class that in both brown trout and Atlantic salmon is predominantly infested by the larvae of freshwater pearl mussel^31–33^. Fish had not previously been in contact with mussel larvae, excluding effects of immunity^39^ and were measured for total length (±1 mm) and wet body weight (±0.1 g). Total length of fish did not differ among salmon-mussel and trout-mussel treatments for Atlantic salmon (ANOVA: F_1,852_ = 2.6, P = 0.11) or landlocked brown trout (ANOVA: F_1,138_ = 2.0, P = 0.16). The difference between salmon-mussel and trout-mussel treatments in anadromous brown trout total length was statistically significant (ANOVA: F_1,939_ = 7.3, P = 0.007), but length differences were small (salmon-mussel: 62.6 ± 5.4 mm; trout-mussel: 63.6 ± 6.1 mm; mean ± sd). Fish husbandry followed standard procedures, and fish were fed commercial fish food pellets. *River Svinesbekken* mussels had fully developed larvae at the time of collection and some mussels may have released larvae during transport to the research station. However, mussels were immediately placed into experimental tanks together with the water they had been transported in, including any potentially released larvae. Larvae release in the wild for *River Figgjo* and *River Flotåna* (B.M.L., unpublished data) suggests that *River Figgjo* mussels were latest among the studied populations in releasing larvae, but that all mussels had released larvae by the end of August.

#### Quantification of infestation

Host specificity may be expressed during the attachment and encystment of larvae on the gills, but also at later stages, when larvae detach prematurely^30^. We therefore recorded infestation at four occasions, over a period of 15 weeks (21 September, 20 October, 29 November 2006 and 5 January 2007). On the first and second occasions, two Atlantic salmon and two anadromous brown trout were randomly sampled from each tank. Landlocked brown trout were not sampled on the first and second occasions because few individuals were used in each tank. On the third occasion, five fish of each type were sampled. On the last occasion, two Atlantic salmon, two anadromous brown trout and either one or two landlocked brown trout were sampled from each tank. In total, 129 landlocked brown trout (out of 140), 219 anadromous brown trout (out of 941) and 219 Atlantic salmon (out of 854) were sampled and inspected for infestation.

Average proportions of fish that died during the four-month experiment per experimental tank were 0.09 ± 0.10 for Atlantic salmon, 0.04 ± 0.04 for anadromous brown trout and 0.07 ± 0.10 for landlocked brown trout (all mean ± SD, N = 20), excluding nine individuals (<0.01% of all fish) that died but could not be assigned to host-type or experimental tank. Mortality rates did not differ between salmon-mussel and trout-mussel treatments for Atlantic salmon (t-test: t = 1.4, P = 0.18, N = 10), anadromous brown trout (t-test, t = 1.0, P = 0.32, N = 10) or landlocked brown trout (t-test, t = 1.4, P = 0.19, N = 10). Fish that died were not inspected for larvae infestation. Our results on the difference in host specificity between salmon-mussel and trout-mussel are robust to the minor effects host mortality may have had on the proportions of infested fish.

Sampled fish were immediately sacrificed and stored in formalin. Before counting of encapsulated larvae, all fish were again measured for total length and weight (see above). Encapsulated larvae were counted on the four left gill arches. The gills were dissected, and larvae were counted under a stereo microscope^30^. When no larvae were found, we also examined the right gills for larvae. In analyses of prevalence (proportion of fish infested), individuals were treated as infested when larvae were found on left and/or right gills. In analysis of infestation intensity (number of larvae), counts of larvae on left gills were used, excluding only individuals that had no larvae on either left or right side. Some individual fish (8 out of 567) were therefore treated as infested with an infestation intensity of zero larvae.

Mussel larvae length was measured for five larvae on all sampled fish (all larvae were measured for fish infested by fewer than five larvae). Larvae were examined under a microscope and length measured with the help of a grid.

#### Statistical analysis

Our main results on the infestation of Atlantic salmon versus brown trout within mussel populations were clear-cut and statistical tests of inference were not performed. Differences in infestation and size of landlocked brown trout versus anadromous brown trout were tested as within trial (i.e. tank) differences, using a Wilcoxon signed rank test when pair differences were non-normally distributed. Those analyses were limited to the third sampling occasion (29 November), because on the other occasions, fish were stored together (pooled across tanks) for each mussel population. Statistical analysis was carried out in R^40^.

## Supplementary information

Supplementary material

## Data Availability

The datasets generated during and/or analysed during the current study are available from the corresponding author on reasonable request.
