# Supplementary material for: Host specificity drives genetic structure in a freshwater mussel
Source: Sci Rep. 2019 Jul 18;9:10409. doi: 10.1038/s41598-019-46802-8 (PMC6639377; doi:10.1038/s41598-019-46802-8)
Supplement: Supplementary file 1 — Supplementary material [file 41598_2019_46802_MOESM1_ESM.pdf]

Supplemental Information for:

Host specificity drives genetic structure in a freshwater mussel

Sebastian Wacker, Bjørn Mejdell Larsen, Sten Karlsson, Kjetil Hindar

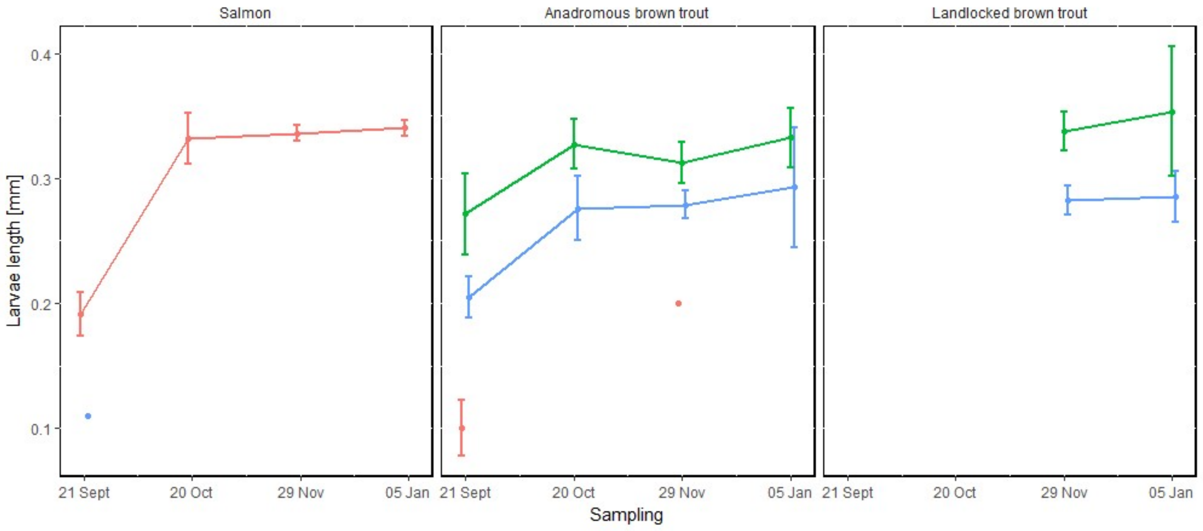

Fig. S1: Size of freshwater pearl mussel larvae at four sampling occasions in an experiment on host specificity, infested on three different host fishes (salmon, anadromous brown trout, landlocked brown trout) and from three different mussel populations (red = *Figgjo* ("salmon-mussel"), green = *Flotåna* ("trout-mussel"), blue = *Svinesbekken* ("trout-mussel")). Means were first calculated across larvae infested on each fish and dots show means across fish, with error bars showing 95% CI. Sample size per host fish, sampling occasion and mussel population ranged from three to 50 individual fish. For salmon infested by trout-mussels and vice-versa, only a single fish was infested in two instances and no error bars are shown. Data points are slightly offset to improve readability.

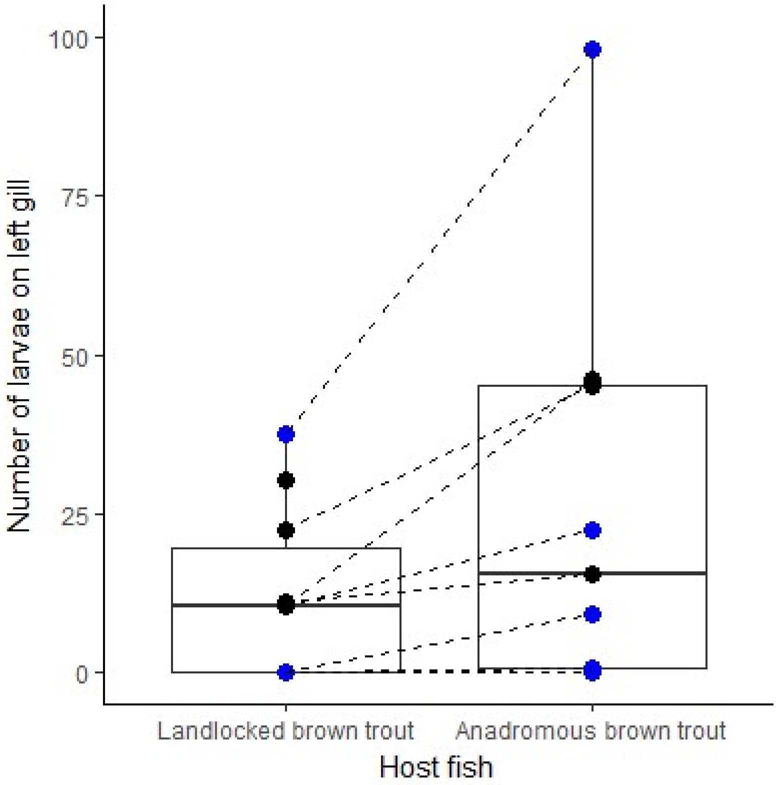

Fig. S2. Infestation (number of larvae on left gill) of landlocked brown trout and anadromous brown trout by freshwater pearl mussel larvae from “trout-mussel” populations at the third sampling day (29 November). Dots indicate mean infestation success per mussel from two populations (*Flotåna* [blue dots], *Svinesbekken* [black dots]) and dashed lines indicate differences in infestation success by larvae from individual mussels.

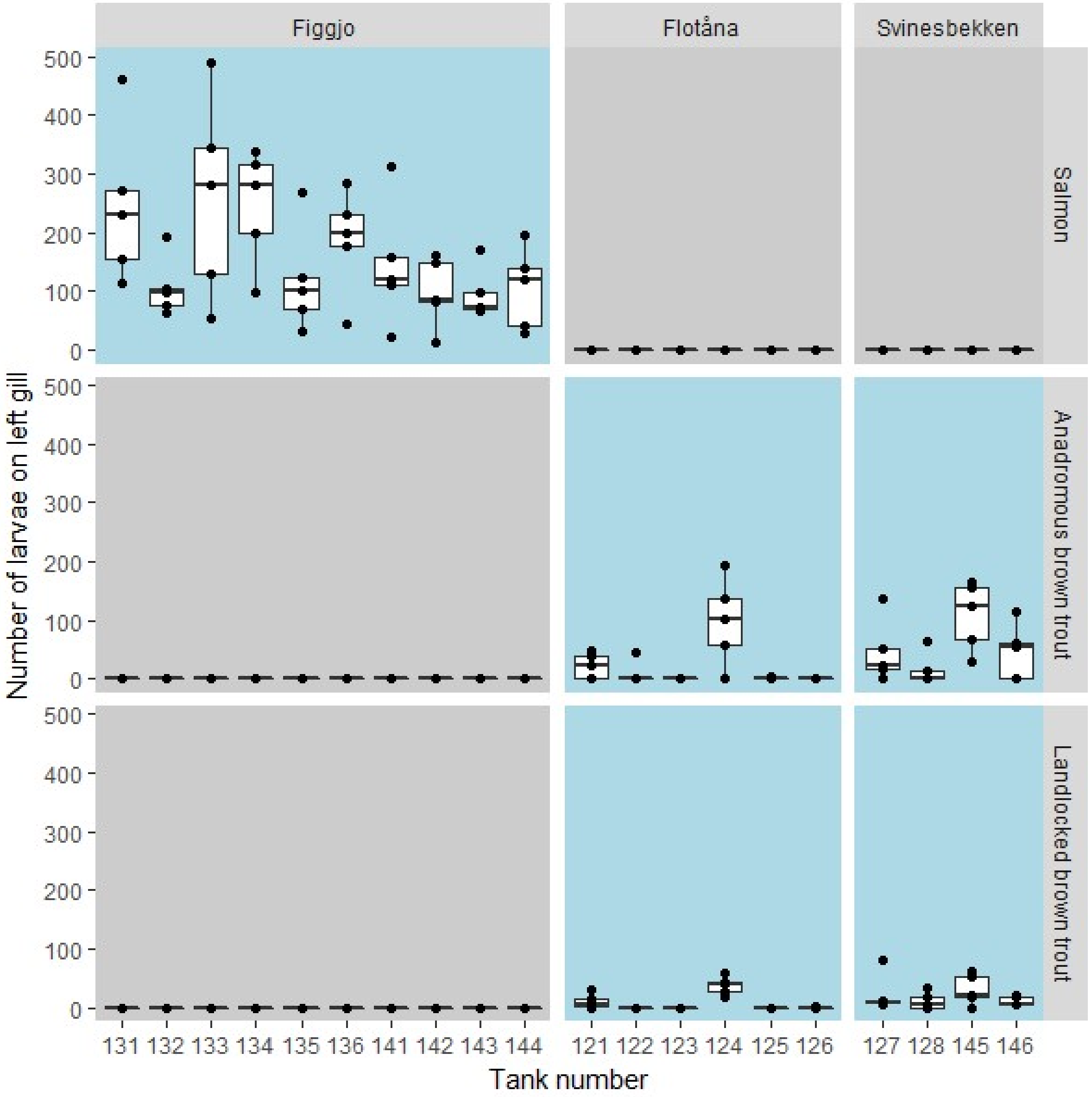

Fig. S3: Infestation (number of larvae on left gills) of host fishes at the third sampling day (29 November), per mussel population (*Figgjo*, *Flotåna* and *Svinesbekk*) and host type (salmon, anadromous brown trout, landlocked brown trout). Data are shown per experimental tank (i.e. individual mussel), with each data point indicating (the number of larvae on) an individual fish. Box plots show medians, upper/lower quartiles, minima and maxima. Sample size is N = 5 per tank and host type.
